# DHEAS and Human Development: An Evolutionary Perspective

**DOI:** 10.3389/fendo.2020.00101

**Published:** 2020-03-03

**Authors:** Benjamin Campbell

**Affiliations:** Department of Anthropology, University of Wisconsin-Milwaukee, Milwaukee, WI, United States

**Keywords:** brain, DHEAS, growth and development, human evolution, middle childhood

## Abstract

Adrenarche, the post-natal rise of DHEA and DHEAS, is unique to humans and the African Apes. Recent findings have linked DHEA in humans to the development of the left dorsolateral prefrontal cortex (LDPFC) between the ages of 4–8 years and the right temporoparietal junction (rTPJ) from 7 to 12 years of age. Given the association of the LDLPFC with the 5-to-8 transition and the rTPJ with mentalizing during middle childhood DHEA may have played an important role in the evolution of the human brain. I argue that increasing protein in the diet over the course of human evolution not only increased levels of DHEAS, but linked meat consumption with brain development during the important 5- to-8 transition. Consumption of animal protein has been associated with IGF-1, implicated in the development of the adrenal zona reticularis (ZR), the site of DHEAS production. In humans and chimps, the zona reticularis emerges at 3–4 years, along with the onset of DHEA/S production. For chimps this coincides with weaning and peak synaptogenesis. Among humans, weaning is completed around 2 ½ years, while synaptogenesis peaks around 5 years. Thus, in chimpanzees, early cortical maturation is tied to the mother; in humans it may be associated with post-weaning provisioning by others. I call for further research on adrenarche among the African apes as a critical comparison to humans. I also suggest research in subsistence populations to establish the role of nutrition and energetics in the timing of adrenarche and the onset of middle childhood.

## Introduction

Adrenarche, the post-natal rise in androgen production by the adrenal gland, including both (DHEA) dehydroepiandrosterone and its sulfated form (DHEAS), has attracted increasing attention for its role in middle childhood (1). Once thought to be involved in the initiation of puberty (2), it is now clear that the rise in DHEAS is an independent event (3). Once thought unique to humans, it now known to be shared with chimps (4), bonobos (5), and gorillas (6). Previously considered for its actions as sex steroid, or a sex steroid precursor (7), DHEAS it is now known to act through a variety of non-genomic mechanisms (8). Yet the functional significance of adrenarche remains little understood.

Together DHEA and DHEAS are the most abundant hormone in human circulation, with DHEAS the more abundant of the two (9). DHEA is generally considered the active form, but DHEAS can be converted to DHEA within cells (7). In addition, DHEAS is the form produced by the adrenal gland (10) making it a marker of adrenal function. Thus, in what follows I will use DHEA/S as a general term, but will differentiate between DHEA and DHEAS where their specific effects have been demonstrated.

In fact, DHEA has a wide variety of physiological effects, including promoting immune function (11), and endothelial function (12), as well as altering brain function (13). At the organismal level DHEA, has documented effects on brain development (14–16), sexuality, mood and cognition (17), cardiovascular disease (18, 19), stroke (20), and mortality [(21, 22), but see (23)]. More recently, DHEA has been linked to follicular development (24–26), with some studies implicating the impact of DHEA on mitochondrial function (27, 28).

Ironically, the wide-ranging physiological impact of DHEA/S makes it hard to focus on a single primary function and has impeded a more systematic understanding of this important hormone. In addition, the most common animal models, the rat and the mouse do not exhibit adrenal production of DHEA/S. However, both mice and rats produce DHEAS within the brain (29), as do humans (30–32). Hence the term neurosteroid for DHEA/S (33). However, neural production of DHEA/S can't explain the origins of adrenal production of DHEA/S and its circulation throughout the body.

More recently, the spiny mouse has been reported to develop an adrenal zona reticularis from post natal day 8–20, a possible analog to human adrenarche, in addition to producing DHEAS in the brain throughout life (34). Interestingly, the species also exhibits high level of fetal adrenal production and menstruates (35) suggesting that it may be a useful rodent model for studying the effects of DHEA/S in humans.

Thus, current results beg the question of what is evolutionarily novel and adaptive about the high and increasing level of circulating DHEAS in humans at the onset of middle childhood around 6–7 years. I suggested that the primary effect (among others) of increasing DHEAS is the maintenance of plasticity in the developing brain (36, 37), a point since elaborated by others (38, 39). Recent finding showing an impact of DHEA on cortical development and cortical-limbic connectivity in children (14–16, 40–43) provide strong support for this argument.

Here I extend my argument about the evolutionary origins of adrenarche in humans to include both the social and nutritional context of the infancy—early child transition as well as the shift from early childhood to middle childhood. More specifically, I suggest that the higher titers of DHEA/S in humans relative to apes may reflect increasing levels meat in the diet with advent of the genus Homo (44). Consumption of meat in early hominids is generally agreed to provide increased energy to support a larger brain, whether this includes a reduction in gut size (45), or not (46), and regardless of when the role of cooking became important (47).

Importantly, consumption of animal protein intake has been related to increased IGF-1 levels in human adults (48, 49) and children (50, 51) and mice (19). At the same time IGF-1 has been implicated in the development of the zona reticularis (52) the layer of the adrenal gland responsible for DHEA production.

I hypothesize that increased meat consumption over the course of human evolution played a role in increasing production of DHEA/S as a part of the extended development of an energetically costly brain (53). Starting with the genus Homo meat consumption would have lead to increased IGF-1 and elevated DHEA/S levels beyond those in the African apes. After weaning, meat provided by males (54) would have provisioned children (55, 56) and supplied protein and energy for cortical synaptogenesis (53, 57). Increased DHEA/S would have acted as a co-factor in promoting cortical maturation, including in the right temporal parietal junction (rTPJ) leading to increased capacity for mentalizing and perspective-taking before the onset of reproductive maturation, a useful trait for a species with pair-bonding and biparental care (58).

I forward this argument in three parts. In the first section, I lay out a series of steps potentially linking intake of animal protein to brain development. These start with the consumption of animal protein which has been associated with higher IGF-1 levels in adults (48, 49) and children (59). IGF-1 also has been implicated in the development of zona reticularis (52), suggesting that higher IGF-1 titers could promote a thicker zona reticularis and increased DHEAS production. IGF-1 itself plays a key role in brain development, both *in utero* and post-natally (60, 61), promoting neurogenesis, neurite growth, and synaptogenesis (62, 63). DHEA has been show to increase energy available in neurons through mitochondrial respiration (64–66), potentially providing additional energy for the metabolic costs of early brain development (53).

In the second section I compare the timing of adrenarche in humans and the great apes. I present evidence showing that while the zona reticularis begins to mature around the age of 3–4 years in both humans (67) and chimpanzees (68), its relationship to other developmental landmarks varies between the two species. Most importantly, in chimpanzees the onset of weaning and peak synaptogenesis in the prefrontal cortex at 3–4 years (69) occur roughly in concert with the emergence of the ZR. In comparison, in humans weaning is completed around 2 ½ years (70, 71), while the peak of synaptogenesis is around 5 years (72) years. Thus, in chimpanzees, early cortical maturation is tied to weaning, while in humans it is more closely associated with DHEAS production.

In the third section, I integrate the timing of DHEA impacts on cortical thickness (14) with social and behavioral markers to present a picture of DHEAS's potential role in the evolution of human childhood stages. Most obviously, the 5-to-8 transition to middle childhood, referred to as the “age of reason and responsibility” by White (73), maps onto the effects of DHEA on LDLPC from 4 to 8 years. Lancy and Grove (74) point out that in many societies children are considered incapable of learning before this age, consistent with the importance of LDLFC for the development of executive function (75).

The impact of DHEA on the rTPJ during middle childhood and its consequences for mentalizing is harder to interpret. Hrdy (76) emphasizes the importance of child care by girls, as practice for their own infants, during human evolution. Mentalizing is an important part of such skills (77, 78). Child care would presumably have been less important for males, but mentalizing may have been useful in learning the skills of cooperative hunting (79). In addition, mentalizing might be beneficial for developing an understanding of one's self in relationship to the opposite sex (1, 80) before puberty exaggerates sexually dimorphic physical and behavioral characteristics that can lead to misunderstandings and tension between the sexes.

Based on the first three sections I end with suggestions for future research directions in the comparative study of adrenarche and middle childhood among both the great apes and humans. Current evidence for adrenarche and its relationship to differences in hormonal (81), cranial (82), and brain (83–85) traits in these two related species (86) is quite limited. A simple direct comparison of adrenarcheal timing in the two species would add immensely to our understanding.

In addition, the onset of middle childhood is thought to be consistent across human populations (74, 87) the evidence regarding adrenarche in subsistence populations (88–91) is scattered and incoherent. A better understanding of variation in adrenarche in the context of poor nutrition, high disease burdens and traditional child care practices would help to ground evolutionary perspectives on adrenarche in a more realistic context. It would also set the foundation of a better understanding of the role of adrenarcheal timing in cognitive development (37).

## IGF-1 and DHEA/S

IGF-1 (Insulin-like Growth Factor 1), as the name suggests, is closely related to insulin. Like insulin, IGF-1 plays a role in glucose regulation (92), and both hormones impact mitochondrial function, protecting against oxidative damage while promoting oxidative capacity (93). However, IGF-1 is most directly associated with protein metabolism, including cell proliferation and differentiation (72) as well as protein synthesis (92). At an organismal level, variation in circulating IGF-1 levels has been related to differences in protein consumption across the life cycle, including during infancy (94), childhood (59), and adulthood (48, 49). In fact, early protein consumption may lead to programing of the IGF-1 axis that becomes apparent during adolescence (95).

IGF-1 is of special interest because it plays an important role in the development of the adrenal gland, including the zona reticularis (52, 96). The zona reticularis develops as primordial stem cells located at the surface of the adrenal cortex move from the zona glomerosa through the zona fasicularis to their final residence in the zona reticularis (52, 96). IGF-1, given its ability to inhibit apoptosis (97) is thought to enhance the survival of the migrating cells. Higher levels of IGF-1 during the formative period of the adrenal gland before the age of 6 years when the zona reticularis is established (67) may lead to increased numbers of progenitor cells reaching the ZR. More ZR cells would presumably lead to increased DHEA/S production. Thus accelerated early somatic growth, linked to increased IGF-1 (98, 99) may be associated with greater DHEA/S as well.

DHEA is generally classified as a weak androgen, i.e., a sex steroid. However, DHEA acts on a variety of cells types and receptor making it more difficult to characterize a single mode of action. For instance in prostate-derived LNCaP tumor cells, DHEA acts at the androgen receptor (AR) and beta estrogen receptor beta (ER-beta) at similar affinities, but the effects at ER-beta appear to be more physiologically relevant (100). In contrast, DHEA does appear to have demonstrable effects on AR mRNA expression in ovarian granulosa cells (101). Thus, the actions of DHEAS are unlikely to be sufficiently characterized as that of a weak androgen alone, and it is important to consider the specific tissue and/or organ involved.

DHEAS has also been suggested to act primarily as a sex-steroid precursor because of its conversion into testosterone and/or estrogen within peripheral target tissues (102). However, the importance of peripheral conversion of DHEAS is most obvious in post-menopausal women for whom ovarian steroid production has ceased. In this case, DHEAS is responsible for 100% of estrogens and 70% of circulating androgens (7). In men and premenopausal women gonadal sources of estrogen and testosterone may obscure the contribution of peripheral conversion of DHEAS to the sex hormones.

More recently, non-genomic mechanisms of DHEA action have been clearly demonstrated, including actions through the IGF-1, sigma 1, TrkA, and GABA receptors as well as the DHEA specific GCPR (8, 103). I specifically mention Sigma-1, TrkAR, and IGF1R because of their role in the brain. The sigma-1R is a chaperone that brings molecules from the cell membrane to the mitochondrial associated membrane (MAM) at the nexus of the mitochondria and endoplasmic reticulum (ER) (103). Sigma-1R has been related to axonal guidance and dendritic arborization (104). The TrkA receptor is important in transducing the effect of nerve growth factor (NGF) and has been related to neuronal differentiation and survival (105). As already mentioned the IGF-1R is related to mitochondrial energy production in neurons (106).

Regardless of the specific receptors involved, DHEA/S has demonstrable effects in the brain. DHEAS regulates the IGF-1 system in the rat hypothalamus by down regulating IGF-1 levels (107). DHEAS is also known to modulate the release of neurotransmitters such as GABA, 5-HT, glutamate and dopamine [see (108) for a review], effects that may involve the Sigma-1R. DHEA, on the other hand, has also been shown to modulate glucose and lactate uptake (109), glucose metabolism (110) and increase mitochondrial energy production in the rat brain (65, 66). A fuller picture of the metabolic effects of DHEA/S' on the brain await future research.

Nonetheless, DHEAS-related impact on neurotransmitter production and release has important implications for patterns of neural activity and brain development. DHEA administration in adults has been shown to inhibit connectivity between the amygdala, the hippocampus and the insula (13) regions connected by glutamatergic and dopaminergic pathways. In addition, individual variation in DHEA from the age of 4–23 years has been linked to differences in connectivity of the amygdala with both the anterior cingulate cortex and the visual cortex (14, 16). Such differences in connectivity may reflect the impact of increasing levels of DHEA on neurotransmitter release and the subsequent production and maintenance synaptic connections.

**Figure 1:**
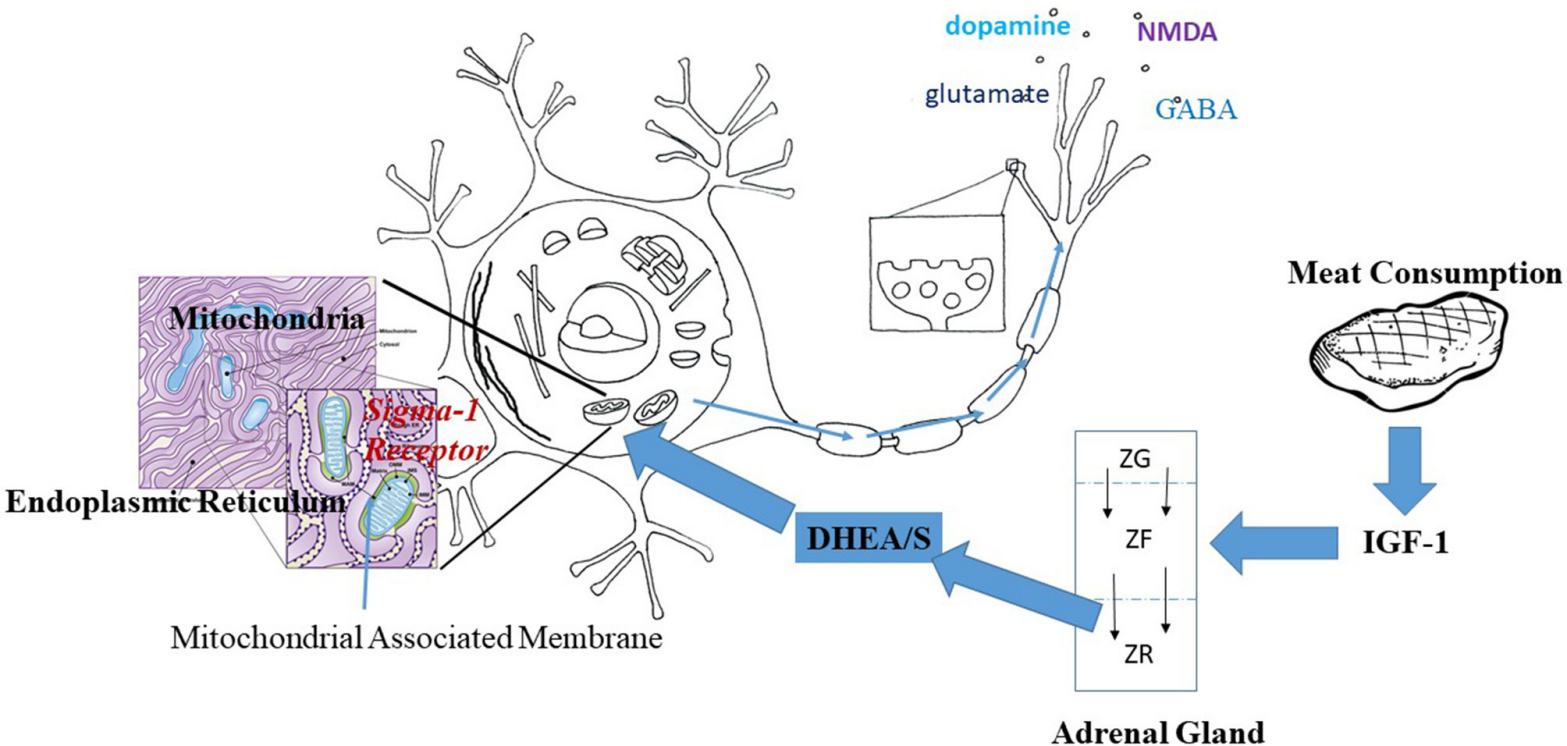
Hypothesized pathway linking meat consumption and neuronal activity. IGF-1 increases with the consumption of animal protein. Within the adrenal gland, IGF-1 prevents apoptosis of cells thus increasing the centripetal movement of cells from the Zona Glomerulus (ZG) and Zona Fascularis (ZF) into the Zona Reticularis (ZR). Increased IGF-1 would result in a thicker ZR and greater production of DHEA/S. DHEA/S crosses the blood brain barrier and enters into neurons. Within the neuron, DHEA acts at the Sigma-1 Receptor located on the mitochondrial associated membrane between the mitochondria and the endoplasmic reticulum. Activation of the Sigma-1 receptor acts to increase energy production and alleviate stress-related production of oxygen free radicals. The result is the increased production and release of neurotransmitters as suggested by the arrows along the axon.

Taken as a whole, findings on DHEA and the Sigma-1R, TrkAR, and IGF-1Rs in neurons suggest that DHEA/S may be one thread in a non-genomic metabolic pathway linking protein intake and brain development in humans, as follows. Increased protein intake would lead to increased IGF-1 production by the liver. Increased IGF-1 would act directly to increase mitochondrial energy production within brain neurons. At the same time, increased levels of IGF-1 during development would promote the growth of the adrenal zona reticularis and with it DHEA/S production. DHEA/S would act on the brain to augment mitochondrial energy production (65, 66) while protecting neurons against related mitochondrial production of oxygen free-radicals and apoptosis (64), with the net income of increasing neurotransmitter release. See Figure 1 for a diagrammatic representation of how meat consumption might play a role in neurotransmitter release acting through the sigma-1 receptor.

## Adrenarche in the African Apes

DHEAS produced by the fetal adrenal is present prenatally in a wide variety of primate species (111). The fetal adrenal is very large *in utero* and then atrophies after birth, meaning DHEAS levels decline rapidly post-natally, with levels among adults generally low across primate species (112). In rhesus macaques, close examination of the adrenal gland indicates that the zona reticularis develops during the period just after birth while the fetal zone of the adrenal is atrophying (113), leading to a transient post-natal increase in DHEAS production (114).

Many primate species, including rhesus and pigtailed macaques and yellow baboons (115) show detectable levels of circulating DHEAS post-natally (112). In fact, across primate species circulating adult DHEAS levels are strongly related to life span (115, 116). Interestingly in the common marmoset (Callithrix jacchus) female show increased levels of DHEAS during adulthood, while adult males do not (117, 118).

However, clear and sustained post-natal increases in DHEAS are limited to humans (119, 120) chimps (4), and bonobos (5). In fact, DHEAS levels appear to be much higher in chimps and bonobos compared to gorillas, which would make adrenarche a derived trait <10 million years old (121). At the same time, substantially higher levels of DHEAS have been documented in human females relative to chimpanzees (68), suggesting that the human pattern is derived from that of chimpanzees and bonobos.

The timing of increases in DHEA/S production across humans, bonobos and chimpanzee species appear to be generally similar. Recently published longitudinal results based on 53 wild chimpanzees from birth to 20 years of age show that urinary DHEAS, after declining from birth, starts to rise around 2–3 years (4). This rise continues until at least 20 years of age, with no significance in urinary DHEAS between the sexes. These findings are consistent with those from a cross-sectional study of 86 captive chimpanzees, ages 1–12 years (122) in which females showing higher serum levels of DHEA starting at 2–4 (122). Males from the same study showing increases in DHEAS starting at 4–6 years.

In humans, a recent cross-sectional study of almost 2,000 (*m* = 1,031; *f* = 926) individuals using a sensitive LC-MS/MS system demonstrate an increase in serum DHEA (119) starting at 3–5 years and continuing into the 20's. Remer et al. (120) show a similar pattern based on LC-MS/MS measurements of urinary DHEA & metabolites in 400 children 3–17 years of age. The data on age patterns of DHEAS for bonobos is much more limited. A single cross-sectional study of 53 captive animals, ages 1–18 years, shows an increase in urinary DHEA-S after 5 years of age (5). As with chimpanzees the values appear to be still increasing at the upper end of the age range.

Taken together, these studies suggest very similar patterns of DHEAS in humans, bonobos and chimpanzees, with onset around 3–5 years and continued increase into at least the late teens. Comparison of age related cortisol patterns re-enforces the similarity of adrenal development between of humans and chimpanzees. In humans, urinary cortisol declines from birth and then starts to increase at 10 years of age with higher levels for males (123). Sabbi et al. (4) show a similar pattern for the wild chimpanzees with an increase from 10 years, but no sex differentiation. Comparable results are not available for bonobos.

It is important, however, to point out that the phenotypic signs used to define adrenarche (presence of acne, body odor, and hair) appear on average at about 8 years for girls (124) and 9 years for boys (125). Phenotypic signs of adrenarche have not be been characterized in either of the two ape species. Thus, while chimpanzees show similar pattern of age related DHEAS and cortisol to humans, the timing of adrenarche as defined by phenotypic markers and associated behavioral changes in chimps and bonobos remains to be investigated.

### Current Research on Adrenarche in Subsistence Populations

Up to this point, I have drawn from results among WEIRD (white educated, industrialized rich and democratic) populations (126) to characterize human adrenarche. However, these populations are generally well-nourished, largely sedentary, and with low disease burdens leaving abundant energy to fuel the development of the brain, confounding any comparison with wild chimpanzees. Nonetheless, Shi et al. (127) report that animal protein intake and fat mass explained a small but significant amount of variation in adrenal androgen secretion (5 and 1% respectively) in a sample of 137 pre-pubertal (ages 3–12 years) German children. Thus, given the existence of undernutrition, high disease burdens and habitual physical activity we might expect that adrenarche, like puberty, would be delayed in subsistence populations. Furthermore, given energetic constraints, the relationship between DHEA/S, fat depots and animal protein intake might be more sharply drawn.

A previous set of studies in subsistence populations based on older sample collection and assay techniques (88, 91, 128) could not adequately address the timing of adrenarche. These studies conceptualized adrenarche as a part of puberty and as a consequence didn't sample individuals young enough to be able catch adrenal onset.

However, three recent studies yield results speak to variation in the timing of adrenarche in subsistence populations and its possible causes. In the study most comparable to that of wild chimps, Helfrecht et al. (89) compared cross-sectional age-related patterns of DHEAS and cortisol derived from hair among Aka pygmies and Ngandu farmers of the Congo with those of Sidama agriculturalist from Ethiopia. The authors used GM models to determine the transition point at which DHEAS levels start to increase again after declining from birth, based on 160 individuals (80 m; 80 f) ages 3–18 years from any all three populations. Results indicate an average age of adrenarche of about 8 years for the Ngandu, and Sidama and 9 years for the Aka, with no significant sex differences in any of the three groups. In addition, age patterns of cortisol among Aka females and Sidama males appear to include a nadir around the age of 10 years.

Importantly, all three of these subsistence populations exhibit high rates of growth stunting (89). Stunting has been associated with lower DHEAS among children in a single study from rural Malawi (59), so undernutrition alone might account for later age at adrenal onset among Aka, Ngandu and Sidama children. In addition, the Aka and the Ngandu, as residents of a tropical forest, presumably carry high parasite burden (89), which could play a role in adrenarche time relative to the non-forest dwelling Sidama.

However, Hlefrecht et al. (89) don't include anthropometric measures to test the relationship of DHEAS with stunting or other nutritional status indices. No do they have measures of animal protein consumption that might be used to test the hypothesis that meat consumption is important to variation in in adrenarcheal timing. Thus, the results are tantalizing, but without reference values for hair DHEAS from industrialized populations for comparison as well as measures of dietary intake and nutritional status they simply beg further investigation.

In a study of the impacts of migration on reproductive maturation (also cross-sectional), Houghton et al. (124) compared the timing of adrenarche in girls among British natives, Bangladeshi natives and both 1st and 2nd generation Bangladeshi immigrants to the U.K., using salivary DHEAS. Average age at adrenarche (determined by a Wiebell regression as a measures of estimating when 50% of the sample passed a threshold value of 400 pg/ml) was 7.1, 7.2, and 7.4 years for the British, Bangladeshi Natives, and 2nd generation immigrants, respectively, while for 1st generation immigrants the average was 5.3 years.

Houghton et al. also analyzed their DHEAS results with regard to nutritional status, reporting that BMI quartiles predicted onset of adrenarche, with the highest quartile showing a significant difference from the others. However, BMI quartiles did not predict DHEAS levels subsequent to adrenarche, suggesting that earlier onset of DHEAS is not related to higher levels of DHEAS during middle childhood and adolescence. In other words, the earlier emergence of the zona reticularis did not appear to be related to the development of a thicker zona reticularis, as indexed by DHEAS production, contrary to my argument above.

The population comparison in Houghton et al.'s study is instructive in two important ways. An earlier study, based on serum DHEAS, reported an average age of adrenarche of 7.7 years among school girls in Taiwan (129). Thus, the timing of adrenarche among the British, Bangledeshi and Taiwan natives provides a clear baseline for the onset of adrenarche among adequately nourished girls at 7–8 years. The results from the Bangledeshi immigrants also shows that adrenarche can vary substantially across populations. On the other hand, the results do not suggest a clear reason for substantially earlier onset of adrenarche among the 1st generation migrants nor do they speak to adrenarche in boys.

Hodges-Simeon et al. (90) investigated the relationship of salivary DHEAS with energetic status in a sample of 90 boys and 81 girls, aged 8–23 years, from the Tsimane of lowland Bolivia, a horticultural/foraging population,. They found higher salivary DHEAS associated with greater superiliac skinfolds and grip strength, among the males, but not the females. The youngest participants in Hodges-Simeon et al.'s sample were 8 years old so estimating adrenarcheal timing was not possible. In fact, 8–23 years is more reflective of pubertal than adrenarcheal changes. Thus, the reported relationship of DHEAS with energy stores and grip strength may be more reflective of the effects of testosterone during puberty, as reported by Campbell and Mbizvo (130) among 441, 12–18 year old, Zimbabwe school boys.

Together these three studies provide clear evidence that the timing of adrenarche can vary across populations and may be related to nutritional status. However, they say little about potential causes for such differences, including differences in energy stores (131), diet (132), and/or heavy parasite burden (133), or protein consumption as proposed here, that characterize subsistence populations. They also leave open potential sex differences in adrenarcheal timing, which may be involved in the development of attachment and gender roles (1).

### Comparative Timing of Zona Reticularis, Brain, and Weaning

Examination of adrenal glands from chimpanzees suggest the ZR begins to emerge around the age of 3 years and continues to broaden into adulthood (134), similar to the pattern found in humans (67). Unfortunately, data regarding the maturation of the zona reticularis is not available for bonobos.

The emergence of the zona reticularis in chimpanzees appears co-incident with the eruption of the deciduous (baby teeth) M1 molar at 3.3 years of age in both wild and captive animals (135, 136). Deciduous M1 eruption is of specific interest because it is related to the age of weaning across mammals in general (137). At the same time a period of elevated synaptogenesis lasts from 3 to 5 years (69) paralleling the process of weaning starting around 3 and finishing at ca. 4.5 years (135). Thus it appears that increased synaptogenesis, with its elevated energy requirements (138), takes place while energy availability from breast milk declines, and DHEA/S levels rise.

In humans the emergence of the ZR at age 3 (67) is similar to that seen in chimpanzees (134). However, the relationship of ZR emergence with the timing of molar eruption, the period of elevated synaptogenesis and weaning differ from that observed in the chimpanzee. Deciduous M1 eruption, at 5.5 years, is delayed by a couple of years relative to chimps. The period of peak brain glucose utilization, associated with synaptogenesis, is reached at about 5 years (53, 57). In contrast, age at weaning in natural fertility populations is close to 2 1/2 years, although the data are poor (70, 71).

In all, it appears that in humans the onset of weaning has been accelerated relative to chimps, with delayed dental maturation and a later peak in synaptogenesis. Thus, the human pattern of early development appears to separate the integration of breast-feeding and brain development from its ancestral roots. This comparison makes it clear that breast feeding intervals have been shortened to increase reproductive rates, while at the same time humans show slower growth rates consistent with our extended life spans, a point made previously by Bogin (55, 56), Bogin et al. (139), Kramer (140), and Kramer and Otárola-Castillo (141).

It has been suggested on the basis of deciduous M1 eruption timing that humans would be expected to exhibit weaning somewhere between 5 and 7 years (137). If this were so, weaning in humans would be associated with a period of declining glucose utilization (53) as in chimps. Instead the temporal advance of weaning means that the period of peak glucose utilization and increased synaptogenesis from the age of 4–8 years falls outside of the period of breastfeeding.

## DHEAS and Human Development

The association of DHEA with the development of the LDLPC is notable for two reasons; 4–8 years is a period of elevated glucose utilization (53, 57) and increased synaptogenesis (138). The timing also maps onto the so-called 5-to-8 transition when children begin to develop cognitive skills that allow for great individual independence (142) White (73) point out that children become cognitively and socially capable of carrying out basic social tasks during this period. Among hunter-gatherer groups this shift is associated with increasing play directed toward subsistence activities [see examples in (143)], as well as the care of younger siblings (144).

The association of DHEA with the rTPJ from 7 to 12 years of age is even more striking because it suggests DHEA's importance to the development of theory of mind (ToM). ToM is well-developed in humans [for whom it is associated with activity in the rTPJ (145)], but rudimentary in chimpanzees (146). During middle childhood, the differentiation of thinking about the thought of others, rather than their actions or feelings is associated with the development of the rTPJ (147, 148).

Thus, DHEA appears to play a role in two key transitional periods; that from infancy to early childhood and from early childhood to the juvenile stage or middle childhood. The first of these steps represents a standard transition in mammals from dependent infants to largely self-sufficient juveniles. As such one might expect that the pattern of DHEAS production during this period would differ between human and chimpanzees primarily by magnitude or timing. The second transition, on the other hand, is thought to be unique to humans as is middle childhood itself (55). Hence the role of DHEA in the development of the rTPJ may be a relatively recent phenomenon in evolutionary terms and as such associated with other novel physiological, neurological or genetic differences between humans and both chimps and bonobos.

The dramatic changes in secondary sexual characteristics during puberty brought on by testosterone and estrogen overshadow any of the physical effect of DHEAS, including acne, body odor, oily skin, and body hair [see (37) for a review]. As mentioned earlier DHEA and DHEAS can be converted into testosterone and/or estrogen and act through the AR (100) or the ER (149). So it possible that DHEA contributes to the effects of testosterone and estradiol. However, given the lack of strong affinity for the AR and ER, DHEA/S it is unlikely to have much discernable impact on secondary sexual characteristics during puberty when testosterone and estrogen levels are rising.

Nonetheless, DHEA/S may continue to have effects on brain development throughout puberty. In fact, among prepubertal children, Nguyen et al. (14) found an interaction of DHEA with testosterone on cortical thickness in the right cingulate cortex and occipital pole while Barendse et al. (150) report an interaction of testosterone and DHEA on white matter microstructure. These findings presumably reflect different modes of action for the two hormones, with testosterone acting through the AR while DHEA acts at other receptors, including sigma-1, TrkA, and IGF-1 (103). If so, prolonged cortical maturation starting at 6 years and continuing into the 20's would appear to reflect increasing levels of DHEA/S as a separate but interrelated process with that of the impact of reproductive maturation on brain development.

Less attention has been given to the implications for the end of the steady rise in DHEAS in the 20's (151, 152). One study reported a peak for females about 25 years, with males showing a peak at 30 (152). This timing is roughly consistent with the end of cortical maturation in the 20's as judged by myelination (153–155). Such continued maturation presumably underlies the emergence of young adulthood as a human developmental stage [see (156) for a discussion of young adulthood].

In contrast, myelination in the chimp appears to reach a peak during puberty, around 12 years (157). However, it is unclear how this timing corresponds to age patterns of DHEAS. Bernstein et al. (112) show a peak in serum DHEAS at 10 years of age for a sample they label PAN, i.e., both chimpanzees and bonobos. In contrast, Behringer et al. (5) report increases in urinary DHEAS among Bonobos until at least the age of 20 years, as do Sabbi et al. (4) for chimpanzees.

More precise measures of both age-related DHEA/S and cortical changes in humans are needed to determine if the cessation of significant myelinization and increases in DHEA/S in humans are in fact related. Nonetheless, the current findings are consistent with a role for DHEA in the prolonged development of the human cortex starting at age 6, continuing through puberty and into the 20's. On the other hand, the relationship of the timing of DHEA/S and cortical maturation for chimps and bonobos remains an important question for investigation.

## Evolution of Early and Middle Childhood

Up to this point I have focused on physiological and cellular mechanisms underlying adrenarche and the effects of DHEA on the brain, including increased cortical thickness in the LDLPFC from 4 to 8 years, and the rTPJ from 7 to 12 (14). Together these brain changes underlie the behavioral and cognitive changes of the 5-to-8 transition and middle childhood (1, 37). The evolutionary question is the nature of the selection pressures that created this novel human life stage of early and middle childhood, inserted between infancy and adolescence (55, 56, 139).

Bogin (55) suggests three possible hypotheses for the evolutionary benefits to early and middle childhood against the backdrop of generally longer development in humans. These include; (1) a reproductive and feeding strategy for the mother, (2) a way of eliciting child care for older children, (3) a way of reducing the energetic cost of juvenile children. All of these factors appear to apply to early childhood. While a nursing mother is pre-occupied by a nursing infant, toddlers can look to other adults and old siblings for both food and social interaction, while their small size makes them less expensive to feed.

With the advent of middle childhood the focus of development shifts to the role of socialization and cultural learning for the child itself (87). Hrdy (76) stresses the importance of child care experience during middle childhood for girls among hunter-gather societies, such as the Ju'Hoansi. The development of mentalizing would be especially helpful for such mothers in training, as recent findings documenting changes in the social brain during pregnancy emphasize (158). But girls of this age are also encultured in women's subsistence activities, as well as morality and religion (87).

Boys are solicitous of their younger siblings as well. However, among subsistence societies childhood activities start to become gender specific during the 7–12 year stage, with girls expected to do childcare (144). Thus, among H-Gs, pre-pubertal boys will start to spend more time practicing and playing hunting skills [see (159, 160) for examples]. As part of this, mentalizing would be important for coordinating behavior during a hunt. In fact, mentalizing can include understanding the mind of prey animals [see (161) for such accounts among the San].

In addition, mentalizing would be useful for developing an understanding of one's self in relationship to the opposite sex. Del Guidice (1) has argued that middle childhood is a time when attachment becomes sexually differentiated and the increase in DHEAS stimulate genetically-based sexually related behaviors that will be more fully developed with puberty. The fact that DHEAS has been related to amygdala connectivity and emotion in prepubertal children (40) together with the role of amygdala in human chemosensory processing (162, 163) provides a potential link by which body odor at adrenarche might be related to the emergence of sexual awareness. Specifically, the development of body odor as a function of sebaceous glands (37) may be synchronous with the development of the emotional salience of body odor as a signal.

## Human Evolution, Weaning, and Meat

To be convincing arguments about the evolution of unique human traits like mentalizing (148) or the emergence of a novel life stage like middle childhood (55) require both a physiological substrate and evidence for a phylogenetic precursor. The onset of DHEAS production during early childhood (119, 120), and its impact on brain development during both early and middle childhood (14, 42, 43), together with the emergence of mentalizing from 9 to 11 years (147) fits both criteria. Adrenarche is a physiological process with neurological consequences related to behaviors during middle childhood, and the increase in DHEAS is linked to the development of the adrenal ZR. A similar increase in DHEA/S among chimpanzees provides a phylogenetic precursor, though the behavioral consequences of adrenarche for chimpanzees are unclear.

Evidence for the role of meat in human brain evolution starting with the origins of the genus *Homo* can only come from the hominid evolutionary record. Recent findings based on analysis of barium/calcium isotopic ratios from five Australopithecus Africanus teeth suggest weaning around 1 year (164). Furthermore, the isotopic analysis show cyclic changes in the barium/calcium ratios after apparent weaning suggesting renewed period of milk intake in response to environmental fluctuations, similar to that observed among orangutans.

These finding are important in suggest that provisioning by non-material family members during early childhood development may have started alongside an increase in endocranial volume with Homo Habilis (165). In other words, provisioning of weaned infants with meat would have helped to alleviate seasonal undernutrition, thus promoting growth and survival while allowing for energetically expensive brain development during this period as discussed above for modern humans.

It is generally agreed that increased meat consumption was critical to early Homo adaptations, including increased brain size (166, 167). The importance of specific factors, such as the role of cooking in making meat especially energy rich (47, 168) and timing of the habitual use of fire for cooking is a topic of much discussion (169–171). As are the roles of essential fatty acids (172, 173) and vitamins and minerals (174). I am not arguing that DHEA/S supplants those factors, i.e., this is not a case of endocrinological “newcomer bias” with regard to metabolic regulation (175), but that DHEA/S represents a previously unrecognized pathway promoting increased brain size, one that seems to fits the specific trajectory of human brain development.

## Future Research Directions

Because direct experimentation is not possible, arguments about evolutionary history are by their nature speculative. However, evolutionary arguments can be substantiated by what Wilson (176) referred to as consilience, the jumping together of relevant elements, each subject to empirical investigation, to bear on the central question. In this case, I highlight two related areas where further research can generate empirical results that can then to applied to the interpretation of the fossil record. These are; (1) better characterization of adrenarcheal timing and its association with behavior in the Africa apes; and (2) the role of protein consumption and nutritional status in adrenarcheal timing among human subsistence populations and its potential implications for behavioral and cognitive development.

### Adrenarche in Chimpanzees and Bonobos

For the great apes, given the longitudinal results available from wild chimpanzees (4) the immediate focus of inquiry shifts to a more complete characterization of adrenarche among bonobos. On-going work at the Kokolopori and Luikotali study sites in the Democrative Republic of Congo by researchers at the Max Planck Institute for Evolutionary Anthropology in Leipzig (https://www.eva.mpg.de/primat/research-groups/bonobos/main-page) are ideally positioned to produce results for wild bonobos. The value of such results would be greatly increased by comparison with an on-going project among captive bonobos and chimpanzee also on-going at the Max Planck Institute. Most specifically, the availability of a well-tested urinary IGFBP-3 (IGF binding protein 3) assay, as proxy for IGF-1 (5) would make it possible to test the mechanism put forward here. Does individual variation in IGFBP-3 predict variation in DHEAS and the timing of adrenarche? Do longitudinal increases in IGFBP-3 predict increases in DHEA/S within individuals?

If bonobos show age-related increases in DHEA/S similar to those clearly documented for wild chimpanzees by Sabbi et al. (4), together the two species would represent a common pattern useful as a single comparison for humans. On other hand, if patterns of adrenarche are different, it is possible that DHEAS may have some role in reported differences between chimpanzee and bonobo brains (83–85). This is a topic worthy of investigation in its own right.

### Adrenarche in Subsistence Populations

As discussed previously, current results suggest that adrenarche may be delayed by a year or two in subsistence populations, but the possible causes and potential implications have yet to receive much attention. The hypothesis advanced here, that meat consumption plays a role is adrenarche, can be tested by using measures of animal protein intake as well as skinfold measures as a marker of energy stores as predictors of DHEA/S in a lean subsistence population much as Shi et al. (127) found among children in Germany. If the results show a significant relationship with animal protein intake, controlling for measures of energy stores, the next step is test IGF-1 as a possible mechanism mediating protein consumption and DHEA/S.

The question remains as to whether differences in timing of adrenarche associated with undernutrition have a demonstrable impact on the timing of cognitive processes associated with the 5-to-8 transition and middle childhood. In WEIRD populations, in addition to underlying process of brain development, cognitive development is scheduled by age-related progression through school which plays an important role in entraining attention (177–179). In fact, cross-cultural studies suggest that the timing of the 5-8 transition is consistent across societies (74, 87), implying that underlying brain maturation, including peak glucose utilization (53) and associated synaptogenesis (138) show similar timing across populations.

Thus, a significant delay in adrenarcheal timing would seem mostly like to shift the relative impact of increasing DHEA/S levels away from maturation of the LDLPFC at 4–8 years and toward the maturation of the rTPJ from 7 to 12 years. As a consequence, delayed adrenal timing might bias brain development away from impulse control and decision-making during the 5-to-8 transition and more toward mentalizing during middle childhood.

Such a brain might end up producing a mind characterized more by attention to the thoughts of others than to abstract thoughts and rules, as suggested by reports of important differences in attentional style in subsistence vs. WEIRD populations (178, 180). Furthermore, greater emphasis on mentalizing might lead to metalizing about the thoughts of animals, especially among hunter-gatherers who are so intimate with their prey (181–183). Or, in the words of Hallowell (184), animals might be come be seen as non-human persons.

## Conclusion

Recent evidence that DHEA/S plays a role in the development of the human brain calls for an evolutionary explanation of adrenarche among both humans and the African Apes. I hypothesize that increasing consumption of meat among our hominid ancestors promoted increased IGF-1 leading to increased growth of the adrenal reticularis and increased production of DHEA/S. In turn, DHEA/S may promote mitochondrial energy production critical for synaptogenesis and brain development.

Comparison of the timing of brain development relative to weaning and dental eruption patterns suggests that unlike for chimps, in humans the maximal brain energy demands are not supported by maternal energy stores. Thus, provisioning of young children by kin with meat may have to the increased important of DHEA/S in brain development during the 5-to-8 transition along with the later development of a middle childhood stage.

Results from wild chimpanzees demonstrate age related patterns of DHEAS and cortisol very similar to those displayed in humans, providing a baseline from which to understand how DHEAS may have played a role in childhood development over the course of human evolution. More work is needed to determine adrenarcheal timing among wild bonobos, and whether differences in DHEA/S is related to developmental differences between the two ape species.

In addition, findings among subsistence populations are tantalizing in suggesting delayed age at adrenarche relative to the industrialized world. But the current results are subject to interpretation and specific factors behind apparent delays call for investigation. To test the hypothesis suggested here, collect data on animal protein consumption as well as anthropometric measures of energetic status in energetically constrained populations are needed. Such work may have important implications for understanding the impact of adrenarcheal timing on the development of cognition in subsistence populations and by inference among humans generally.

## Author Contributions

BC was responsible for the entire production of this manuscript.

### Conflict of Interest

The author declares that the research was conducted in the absence of any commercial or financial relationships that could be construed as a potential conflict of interest.

## References

[1] Del Giudice M Middle childhood: an evolutionary-developmental synthesis. Child Dev Perspect. (2014) 8:193–200. 10.1111/cdep.12084

[2] Cutler GB Jr Loriaux DL Adrenarche and its relationship to the onset of puberty. Fed Proc. (1980) 39:2384–90. 6445284

[3] Palmert MR Hayden DL Mansfield MJ Crigler JF Jr Crowley WF Jr Chandler DW. The longitudinal study of adrenal maturation during gonadal suppression: evidence that adrenarche is a gradual process. J Clin Endocrinol Metab. (2001) 86:4536–42. 10.1210/jcem.86.9.7863 11549704

[4] Sabbi KH Muller MN Machanda ZP Otali E Fox SA Wrangham RW. Human-like adrenal development in wild chimpanzees: a longitudinal study of urinary dehydroepiandrosterone-sulfate and cortisol. Am J Primatol. (2019). 10.1002/ajp.23064. [Epub ahead of print]. 31709585 PMC7211130

[5] Behringer V Hohmann G Stevens JM Weltring A Deschner T. Adrenarche in bonobos (*Pan paniscus*): evidence from ontogenetic changes in urinary dehydroepiandrosterone-sulfate levels. J Endocrinol. (2012) 214:55–65. 10.1530/JOE-12-0103 22562655

[6] Edes AN. Dehydroepiandrosterone-sulfate (DHEA-S), sex, and age in zoo-housed western lowland gorillas (Gorilla gorilla gorilla). Primates. (2017) 58:385–92. 10.1007/s10329-017-0602-2 28229260

[7] Labrie F Luu-The V Bélanger A Lin SX Simard J Pelletier G. Is dehydroepiandrosterone a hormone? J Endocrinol. (2005) 187:169–96. 10.1677/joe.1.06264 16293766

[8] Clark BJ Prough RA Klinge CM. Mechanisms of action of dehydroepiandrosterone. Vitam Horm. (2018) 108:29–73. 10.1016/bs.vh.2018.02.003 30029731

[9] Maninger N Wolkowitz OM Reus VI Epel ES Mellon SH. Neurobiological and neuropsychiatric effects of dehydroepiandrosterone (DHEA) and DHEA sulfate (DHEAS). Front. Neuroendocrinol. (2009) 30:65–91. 10.1016/j.yfrne.2008.11.002 19063914 PMC2725024

[10] Rainey WE Nakamura Y. Regulation of the adrenal androgen biosynthesis. J Steroid Biochem Mol Biol. (2008) 108:281–86. 10.1016/j.jsbmb.2007.09.015 17945481 PMC2699571

[11] Alves VB Basso PJ Nardini V Silva A Chica JE Cardoso CR. Dehydroepiandrosterone (DHEA) restrains intestinal inflammation by rendering leukocytes hyporesponsive and balancing colitogenic inflammatory responses. Immunobiology. (2016) 221:934–43. 10.1016/j.imbio.2016.05.013 27263829

[12] Huerta-García E Ventura-Gallegos JL Victoriano ME Montiél-Dávalos A Tinoco-Jaramillo G López-Marure R. Dehydroepiandrosterone inhibits the activation and dysfunction of endothelial cells induced by high glucose concentration. Steroids. (2012) 77:233–40. 10.1016/j.steroids.2011.11.010 22155530

[13] Sripada RK Welsh RC Marx CE Liberzon I. The neurosteroids allopregnanolone and dehydroepiandrosterone modulate resting-state amygdala connectivity. Hum Brain Map. (2014) 35:3249–61. 10.1002/hbm.22399 24302681 PMC4739102

[14] Nguyen TV McCracken JT Ducharme S Cropp BF Botteron KN Evans AC. Interactive effects of dehydroepiandrosterone and testosterone on cortical thickness during early brain development. J Neurosci. (2013) 33:10840–8. 10.1523/JNEUROSCI.5747-12.2013 23804104 PMC3693059

[15] Nguyen TV Gower P Albaugh MD Botteron KN Hudziak JJ Fonov VS. The developmental relationship between DHEA and visual attention is mediated by structural plasticity of cortico-amygdalar networks. Psychoneuroendocrinology. (2016) 70:122–33. 10.1016/j.psyneuen.2016.05.003 27236606 PMC4907862

[16] Nguyen TV Wu M Lew J Albaugh MD Botteron KN Hudziak JJ. Dehydroepiandrosterone impacts working memory by shaping cortico-hippocampal structural covariance during development. Psychoneuroendocrinology. (2017) 86:110–21. 10.1016/j.psyneuen.2017.09.013 28946055 PMC5659912

[17] Pluchino N Drakopoulos P Bianchi-Demicheli F Wenger JM Petignat P Genazzani AR Neurobiology of DHEA and effects on sexuality, mood and cognition. J Steroid Biochem Mol Biol. (2015) 45:273–80. 10.1016/j.jsbmb.2014.04.012 24892797

[18] Mannella P Simoncini T Caretto M Genazzani AR. Dehydroepiandrosterone and cardiovascular disease. Vitam Horm. (2018) 108:333–53. 10.1016/bs.vh.2018.05.001 30029733

[19] Wu TT Chen Y Zhou Y Adi D Zheng YY Liu F. Prognostic value of dehydroepiandrosterone sulfate for patients with cardiovascular disease: a systematic review and neta-analysis. J Am Heart Assoc. (2017) 6:e004896. 10.1161/JAHA.116.004896 28476876 PMC5524067

[20] Jiménez MC Sun Q Schürks M Chiuve S Hu FB Manson JE. Low dehydroepiandrosterone sulfate is associated with increased risk of ischemic stroke among women. Stroke. (2013) 44:1784–9. 10.1161/STROKEAHA.111.000485 23704104 PMC3811081

[21] Phillips AC Carroll D Gale CR Lord JM Arlt W Batty GD. Cortisol, DHEA sulphate, their ratio, and all-cause and cause-specific mortality in the Vietnam Experience Study. Euro J Endocrinol. (2010) 163:285–92. 10.1530/EJE-10-0299 20498139

[22] Ohlsson C Labrie F Barrett-Connor E Karlsson MK Ljunggren O Vandenput L. Low serum levels of dehydroepiandrosterone sulfate predict all-cause and cardiovascular mortality in elderly Swedish men. J Clin Endocrinol Metab. (2010) 95:4406–14. 10.1210/jc.2010-0760 20610590

[23] Ohlsson C Vandenput L Tivesten A. DHEA and mortality: what is the nature of the association? J Steroid Biochem Mol Biol. (2015) 145:248–53. 10.1016/j.jsbmb.2014.03.006 24704256

[24] Ford JH. Reduced quality and accelerated follicle loss with female reproductive aging - does decline in theca dehydroepiandrosterone (DHEA) underlie the problem? J Biomedi Sci. (2013) 20:93. 10.1186/1423-0127-20-93 24330163 PMC3878748

[25] Gleicher N Weghofer A Barad DH. Improvement in diminished ovarian reserve after dehydroepiandrosterone supplementation. Reprod Biomed Online. (2010) 21:360–5. 10.1016/j.rbmo.2010.04.006 20638339

[26] Li J Yuan H Chen Y Wu H Wu H Li L. A meta-analysis of dehydroepiandrosterone supplementation among women with diminished ovarian reserve undergoing *in vitro* fertilization or intracytoplasmic sperm injection. Int J Gynaecol Obstet. (2015) 131:240–5. 10.1016/j.ijgo.2015.06.028 26421833

[27] Lin LT Wang PH Wen ZH Li CJ Chen SN Tsai EM. The Application of dehydroepiandrosterone on improving mitochondrial function and reducing apoptosis of cumulus cells in poor ovarian responders. Int J Medi Sci. (2017) 14:585–94. 10.7150/ijms.18706 28638275 PMC5479128

[28] Lin LT Wang PH Chen SN Li CJ Wen ZH Cheng JT. Protection of cumulus cells following dehydroepiandrosterone supplementation. Gynecol Endocrinol. (2018) 33:100–4. 10.1080/09513590.2016.1214262 27684542

[29] Robel P Young J Corpéchot C Mayo W Perché F Haug M. Biosynthesis and assay of neurosteroids in rats and mice: functional correlates. J Steroid Biochem Mol Biol. (1995) 53:355–60. 10.1016/0960-0760(95)00074-A 7626480

[30] Brown RC Cascio C Papadopoulos V. Pathways of neurosteroid biosynthesis in cell lines from human brain: regulation of dehydroepiandrosterone formation by oxidative stress and beta-amyloid peptide. J Neurochem. (2000) 74:847–59. 10.1046/j.1471-4159.2000.740847.x 10646538

[31] Zwain IH Yen SS. Dehydroepiandrosterone: biosynthesis and metabolism in the brain. Endocrinology. (1999) 140:880–7. 10.1210/endo.140.2.6528 9927319

[32] Guazzo EP Kirkpatrick PJ Goodyer IM Shiers HM Herbert J. Cortisol, dehydroepiandrosterone (DHEA), and DHEA sulfate in the cerebrospinal fluid of man: relation to blood levels and the effects of age. J Clin Endocrinol Metab. (1996) 81:3951–60. 10.1210/jcem.81.11.8923843 8923843

[33] Baulieu EE. Neurosteroids: a novel function of the brain. Psychoneuroendocrinology. (1998) 23:963–87. 10.1016/S0306-4530(98)00071-7 9924747

[34] Quinn TA Ratnayake U Dickinson H Castillo-Melendez M Walker DW. The feto-placental unit, and potential roles of dehydroepiandrosterone (DHEA) in prenatal and postnatal brain development: a re-examination using the spiny mouse. J Steroid Biochem Mol Biol. (2016) 160:204–13. 10.1016/j.jsbmb.2015.09.044 26485665

[35] Bellofiore N Evans J. Monkeys, mice and menses: the bloody anomaly of the spiny mouse. J Assist Reprod Genet. (2019) 36:811–7. 10.1007/s10815-018-1390-3 30610663 PMC6541669

[36] Campbell BC. Adrenarche and the evolution of human life history. Am J Hum Biol. (2006) 18:569–89. 10.1002/ajhb.20528 16917887

[37] Campbell BC. Adrenarche and middle childhood. Hum Nat. (2011) 22:327–49. 10.1007/s12110-011-9120-x 22388879

[38] Greaves RF Wudy SA Badoer E Zachrin M Hirst JJ Quinn T. A tale of two steroids: the importance of the androgens DHEA and DHEAS for early neurodevelopment. J Steroid Biochem Mol Biol. (2019) 188:77–85. 10.1016/j.jsbmb.2018.12.007 30557606

[39] Quinn T Greaves R Badoer E Walker D. DHEA in prenatal and postnatal life: implications for brain and behavior. Vitam Horm. (2018) 108:145–74 10.1016/bs.vh.2018.03.001 30029725

[40] Barendse MEA Simmons JG Byrne ML Patton G Mundy L Olsson CA. Associations between adrenarcheal hormones, amygdala functional connectivity and anxiety symptoms in children. Psychoneuroendocrinology. (2018) 97:156–63. 10.1016/j.psyneuen.2018.07.020 30036793

[41] Ellis R Fernandes A Simmons JG Mundy L Patton G Allen NB. Relationships between adrenarcheal hormones, hippocampal volumes and depressive symptoms in children. Psychoneuroendocrinology. (2019) 104:55–63. 10.1016/j.psyneuen.2019.02.016 30802711

[42] Farooqi NAI Scotti M Lew JM Botteron KN Karama S McCracken JT. Role of DHEA and cortisol in prefrontal-amygdalar development and working memory. Psychoneuroendocrinology. (2018) 98:86–94. 10.1016/j.psyneuen.2018.08.010 30121549 PMC6204313

[43] Farooqi NAI Scotti M Yu A Lew J Monnier P Botteron KN. Sex-specific contribution of DHEA-cortisol ratio to prefrontal-hippocampal structural development, cognitive abilities and personality traits. J Neuroendocrinol. (2019) 31:e12682. 10.1111/jne.12682 30597689 PMC6394408

[44] Ferraro JV Plummer TW Pobiner BL Oliver JS Bishop LC Braun DR. Earliest archaeological evidence of persistent hominin carnivory. PLoS ONE. (2013) 8:e62174. 10.1371/journal.pone.0062174 23637995 PMC3636145

[45] Aeillo LC Wheeler P The expensive tissue hypothesis: the brain and the digestive system in human and primate evolution. Curr Anthropol. (1995) 36:199–221. 10.1086/204350

[46] Navarrete A van Schaik CP Isler K. Energetics and the evolution of human brain size. Nature. (2011) 480:91–3. 10.1038/nature10629 22080949

[47] Wrangham R Catching Fire: How Cooking Made Us Human. New York, NY: Basic Books (2009).

[48] Levine ME Suarez JA Brandhorst S Balasubramanian P Cheng CW Madia F. Low protein intake is associated with a major reduction in IGF-1, cancer, and overall mortality in the 65 and younger but not older population. Cell Metab. (2014) 19:407–17. 10.1016/j.cmet.2014.02.006 24606898 PMC3988204

[49] Giovannucci E Pollak M Liu Y Plaz EA Majeed N Rimm EB. Nutritional predictors of insulin-like growth factor I and their relationships to cancer in men. Cancer Epidemiol Biomark Prev. (2003) 12:84–9. 12582016

[50] Hoppe C Udam TR Lauritzen L Mølgaard C Juul A Michaelsen KF. Animal protein intake, serum insulin-like growth factor I, and growth in healthy 2.5-y-old Danish children. Am J Clin Nutr. (2004) 80:447–452. 10.1093/ajcn/80.2.447 15277169

[51] Rogers IS Gunnell D Emmett PM Glynn LR Dunger DB Holly JM. Cross-sectional associations of diet and insulin-like growth factor levels in 7-to 8-year-old children. Cancer Epidemiol Biomark Prev. (2005) 14:204–12. 15668496

[52] Belgorosky A Baquedano MS Guercio G Rivarola MA. Expression of the IGF and the aromatase/estrogen receptor systems in human adrenal tissues from early infancy to late puberty: implications for the development of adrenarche. Rev Endocr Metab Disord. (2009) 10:51–61. 10.1007/s11154-008-9105-1 18792783

[53] Kuzawa CW Chugani HT Grossman LI Lipovich L Muzik O Hof PR. Metabolic costs and evolutionary implications of human brain development. Proc Natl Acad Sci USA. (2014) 111:13010–5. 10.1073/pnas.1323099111 25157149 PMC4246958

[54] Wood BM Marlowe FW. Household and kin provisioning by Hadza men. Hum Nat. (2013) 24:280–317. 10.1007/s12110-013-9173-0 23813245

[55] Bogin B Evolutionary hypotheses for human childhood. Yearb Phys Anthropol. (1997) 40:63–89. 10.1002/(SICI)1096-8644(1997)25+<63::AID-AJPA3>3.0.CO;2-8

[56] Bogin B. Childhood, adolescence and longevity: a multilevel model of the evolution of reserve capacity in human life history. Am J Hum Biol. (2009) 21:567–77. 10.1002/ajhb.20895 19326459

[57] Chugani HT Phelps ME Mazziotta JC. Positron emission tomography study of human brain functional development. Ann Neurol. (1987) 22:87–97. 10.1002/ana.410220408 3501693

[58] Dunbar RI Shultz S. Evolution in the social brain. Science. (2007) 317:1344–7. 10.1126/science.1145463 17823343

[59] Semba RD Trehan I Li X Salem N Jr Moaddel R Ordiz MI. Low serum ω-3 and ω-6 polyunsaturated fatty acids and other metabolites are associated with poor linear growth in young children from rural Malawi. Am J Clin Nutr. (2017) 106:1490–9. 10.3945/ajcn.117.164384 29070563 PMC5698844

[60] Dyer AH Vahdatpour C Sanfeliu A Tropea D. The role of insulin-like growth factor 1 (IGF-1). in brain development, maturation and neuroplasticity. Neuroscience. (2016) 325:89–99. 10.1016/j.neuroscience.2016.03.056 27038749

[61] Sádaba MC Martín-Estal I Puche JE Castilla-Cortázar I. Insulin-like growth factor 1 (IGF-1) therapy: Mitochondrial dysfunction and diseases. Biochim Biophys Acta. (2016) 1862:1267–78. 10.1016/j.bbadis.2016.03.010 27020404

[62] Joseph D'Ercole A Ye P. Expanding the mind: insulin-like growth factor I and brain development. Endocrinology. (2008) 149:5958–62. 10.1210/en.2008-0920 18687773 PMC2613055

[63] O'Kusky J Ye P. Neurodevelopmental effects of insulin-like growth factor signaling. Front Neuroendocrinol. (2012) 33:2340–51. 10.1016/j.yfrne.2012.06.002 22710100 PMC3677055

[64] Grimm A Schmitt K Lang UE Mensah-Nyagan AG Eckert A. Improvement of neuronal bioenergetics by neurosteroids: implications for age-related neurodegenerative disorders. Biochim Biophys Acta. (2014) 1842:2427–38. 10.1016/j.bbadis.2014.09.013 25281013

[65] Patel MA Katyare SS. Treatment with dehydroepiandrosterone (DHEA) stimulates oxidative energy metabolism in the cerebral mitochondria. a comparative study of effects in old and young adult rats. Neurosci Lett. (2006) 402:131–6. 10.1016/j.neulet.2006.03.057 16630690

[66] Patel MA Katyare SS. Effect of dehydroepiandrosterone (DHEA) treatment on oxidative energy metabolism in rat liver and brain mitochondria. a dose-response study. Clin Biochem. (2007) 40:57–65. 10.1016/j.clinbiochem.2006.08.014 17052700

[67] Dhom G The prepubertal and pubertal growth of the adrenal (adrenarche). Beitr Tahol. (1973) 150:357–77. 10.1016/S0005-8165(73)80086-1 4785066

[68] Blevins JK Coxworth JE Herndon JG Hawkes K. Brief communication: adrenal androgens and aging: female chimpanzees (Pan troglodytes) compared with women. Am J Phys Anthropol. (2013) 151:643–8. 10.1002/ajpa.22300 23818143 PMC4412270

[69] Bianchi S Stimpson CD Duka T Larsen MD Janssen WG Collins Z. Synaptogenesis and development of pyramidal neuron dendritic morphology in the chimpanzee neocortex resembles humans. Proc Natl Acad Sci USA. (2013) 110(Suppl. 2):10395–401. 10.1073/pnas.1301224110 23754422 PMC3690614

[70] Sellen DW. Evolution of infant and young child feeding: implications for contemporary public health. Ann Rev Nutr. (2007) 27:123–48. 10.1146/annurev.nutr.25.050304.092557 17666009

[71] Sellen DW. Evolution of human lactation and complementary feeding: implications for understanding contemporary cross-cultural variation. Adv Exp Medi Biol. (2009) 639:253–82. 10.1007/978-1-4020-8749-3_18 19227547

[72] Liu X Somel M Tang L Yan Z Jiang X Guo S. Extension of cortical synaptic development distinguishes humans from chimpanzees and macaques. Genome Res. (2012) 22:611–22. 10.1101/gr.127324.111 22300767 PMC3317144

[73] White S The child's entry into the Age of Reason. In: Sameroff AJ Haith MH editors. The Five to Seven Year shift: The Age of Reason and Responsibility. Chicago: University of Chicago Press (1996) p. 17–30.

[74] Lancy DF Grove MA. Getting noticed. middle childhood in cross-cultural perspective Hum Nat. (2011) 22:281–302. 10.1007/s12110-011-9117-5 22388877

[75] Smith E Anderson A Thurm A Shaw P Maeda M Chowdhry F. Prefrontal activation during executive tasks emerges over early childhood: evidence from functional near infrared spectroscopy. Dev Neuropsychol. (2017) 42:253–64. 10.1080/87565641.2017.1318391 28622028 PMC8074193

[76] Hrdy S Mothers and Others. (2009). Cambridge: Belknap Press.

[77] Shai D Dollberg D Szepsenwol O. The importance of parental verbal and embodied mentalizing in shaping parental experiences of stress and coparenting. Infant Behav. (2017) 49:87–96. 10.1016/j.infbeh.2017.08.003 28818676

[78] Shai D Belsky J. Parental embodied mentalizing: how the nonverbal dance between parents and infants predicts children's socio-emotional functioning. Attach Hum Dev. (2017) 19:191–219. 10.1080/14616734.2016.1255653 27852170

[79] Whiten A Erdal D. The human socio-cognitive niche and its evolutionary origins. Philos Trans R Soc Lond B Biol Sci. (2012) 367:2119–29. 10.1098/rstb.2012.0114 22734055 PMC3385679

[80] Colle L Del Giudice M Patterns of attachment and emotional competence in middle childhood. Soc Dev. (2011) 20:51–72. 10.1111/j.1467-9507.2010.00576.x

[81] Behringer V Deschner T Deimel C Stevens JM Hohmann G. Age-related changes in urinary estosterone levels suggest differences in puberty onset and divergent life history strategies in bonobos and chimpanzees. Horm Behav. (2014) 66:525–33. 10.1016/j.yhbeh.2014.07.011 25086337

[82] Lieberman DE Carlo J Ponce de León M Zollikofer CP. A geometric morphometric analysis of heterochrony in the cranium of chimpanzees and bonobos. J Hum Evol. (2007) 52:647–62. 10.1016/j.jhevol.2006.12.005 17298840

[83] Issa HA Staes N Diggs-Galligan S Stimpson CD Gendron-Fitzpatrick A Taglialatela JP. Comparison of bonobo and chimpanzee brain microstructure reveals differences in socio-emotional circuits. Brain Struct Funct. (2019) 224:39–51. 10.1007/s00429-018-1751-9 30306256

[84] Rilling JK Scholz J Preuss TM Glasser MF Errangi BK Behrens TE. Differences between chimpanzees and bonobos in neural systems supporting social cognition. Soc Cogn Affect Neurosci. (2012) 7:369–79. 10.1093/scan/nsr017 21467047 PMC3324566

[85] Stimpson CD Barger N Taglialatela JP Gendron-Fitzpatrick A Hof PR Hopkins WD. Differential serotonergic innervation of the amygdala in bonobos and chimpanzees. Soc Cogn Affect Neurosci. (2016) 11:413–22. 10.1093/scan/nsv128 26475872 PMC4769630

[86] Gruber T Clay Z. A comparison between bonobos and chimpanzees: a review and update. Evol Anthropol. (2016) 25:239–52. 10.1002/evan.21501 27753219

[87] Konner N The Evolution of Childhood: Relationships, Emotion, Mind. Cambridge, MA: Belknap Press (2010).

[88] Campbell BC Leslie PW Little MA Campbell KL. Pubertal timing, hormones, and body composition among adolescent Turkana males. Am J Phys Anthropol. (2005) 128:896–905. 10.1002/ajpa.20204 16110484

[89] Helfrecht C Hagen EH deAvila D Bernstein RM Dira SJ Meehan CL. DHEAS patterning across childhood in three sub-Saharan populations: associations with age, sex, ethnicity, and cortisol. Am J Hum Biol. (2018) 30:e23090. 10.1002/ajhb.23090 29226590

[90] Hodges-Simeon CR Prall SP Blackwell AD Gurven M Gaulin SJC. Adrenal maturation, nutritional status, and mucosal immunity in Bolivian youth. Am J Hum Biol. (2017) 29:1–14. 10.1002/ajhb.23025 28653779

[91] Worthman CM Epidemiology of human development In: Panter-Brick C Worthman CM editors. Hormones, Health and Behavior: A Socio-Ecological and Life Span Perspective. Cambridge, MA: Cambridge University Press (1999). p. 47–104. 10.1017/CBO9780511623462.003

[92] Vassilakos G Barton ER. Insulin-like growth factor I regulation and its actions in skeletal muscle. Comp Physiol. (2018) 9:413–38. 10.1002/cphy.c180010 30549022

[93] Puche JE García-Fernández M Muntané J Rioja J González-Barón S Castilla Cortazar I. Low doses of insulin-like growth factor-I induce mitochondrial protection in aging rats. Endocrinology. (2008) 149:2620–7. 10.1210/en.2007-1563 18276748

[94] Wiley AS Joshi SM Lubree HG Bhat DS Memane NS Raut DA. IGF-I and IGFBP-3 concentrations at 2 years: associations with anthropometry and milk consumption in an Indian cohort. Euro J Clin Nutr. (2018) 72:564–71. 10.1038/s41430-018-0108-z 29453428

[95] Switkowski KM Jacques PF Must A Fleisch A Oken E. Associations of protein intake in early childhood with body composition, height, and insulin-like growth factor I in mid-childhood and early adolescence. Am J Clin Nutr. (2019) 109:1154–63. 10.1093/ajcn/nqy354 30869114 PMC6462426

[96] Baquedano MS Belgorosky A. Human adrenal cortex: epigenetics and postnatal functional zonation. Horm Res Paediatr. (2018) 89:331–40. 10.1159/000487995 29742513

[97] Kooijman R. Regulation of apoptosis by insulin-like growth factor (IGF)-1. Cytokine Growth Factor Rev. (2006) 17:305–23. 10.1016/j.cytogfr.2006.02.002 16621671

[98] Giapros VI Schiza V Challa AS Pantou C Theocharis PD Andronikou SK. Serum insulin-like growth factor I (IGF-I), IGF-binding proteins-1 and−3, and postnatal growth of late preterm infants. Horm Metab Res. (2012) 44:845–50. 10.1055/s-0032-1321759 22791601

[99] Iñiguez G Salazar T Roman R Avila A Gunn RD Cassorla F. Effects of the IGF-I/IGFBP-3 complex on GH and ghrelin nocturnal concentrations in low birth weight children. Clin Endocrinol. (2006) 65:687–92. 10.1111/j.1365-2265.2006.02650.x 17054474

[100] Chen F Knecht K Birzin E Fisher J Wilkinson H Mojena M. Direct agonist/antagonist functions of dehydroepiandrosterone. Endocrinology. (2005) 146:4568–76. 10.1210/en.2005-0368 15994348

[101] Hu Q Hong L Nie M Wang Q Fang Y Dai Y. The effect of dehydroepiandrosterone supplementation on ovarian response is associated with androgen receptor in diminished ovarian reserve women. J Ovarian Res. (2017) 10:32. 10.1186/s13048-017-0326-3 28472976 PMC5418866

[102] Labrie F. Intracrinology in action: importance of extragonadal sex steroid biosynthesis and inactivation in peripheral tissues in both women and men. J Steroid Biochem Mol Biol. (2015) 145:131–2. 10.1016/j.jsbmb.2014.09.012 25240498

[103] Prough RA Clark BJ Klinge CM. Novel mechanisms for DHEA action. J Mol Endocrinol. (2016) 56:R139–55. 10.1530/JME-16-0013 26908835

[104] Tsai SA Su TP. Sigma-1 receptors fine-tune the neuronal networks. Adv Exp Med Biol. (2017) 964:79–83. 10.1007/978-3-319-50174-1_7 28315266 PMC6100794

[105] Marlin MC Li G. Biogenesis and function of the NGF/TrkA signaling endosome. Int Rev Cell Mol Biol. (2015) 314:239–57. 10.1016/bs.ircmb.2014.10.002 25619719 PMC4307610

[106] Gazit N Vertkin I Shapira I Helm M Slomowitz E Sheiba M. IGF-1 receptor differentially regulates spontaneous and evoked transmission via mitochondria at hippocampal synapses. Neuron. (2016) 89:583–97. 10.1016/j.neuron.2015.12.034 26804996 PMC4742535

[107] Ribeiro MF Garcia-Segura LM. Dehydroepiandrosterone regulates insulin-like growth factor-1 system in adult rat hypothalamus. Endocrine. (2002) 17:19–134. 10.1385/ENDO:17:2:129 12041914

[108] Pérez-Neri I Montes S Ojeda-López C Ramírez-Bermúdez J Ríos C. Modulation of neurotransmitter systems by dehydroepiandrosterone and dehydroepiandrosterone sulfate: mechanism of action and relevance to psychiatric disorders. Prog Neuropsychopharmacol Biol Psychiatry. (2008) 32:1118–30. 10.1016/j.pnpbp.2007.12.001 18280022

[109] de Souza DK Ribeiro MF Kucharski LC. Effects of dehydroepiandrosterone (DHEA) and lactate on glucose uptake in the central nervous system. Neurosci Lett. (2012) 507:62–6. 10.1016/j.neulet.2011.11.052 22172926

[110] Vieira-Marques C Arbo BD Ruiz-Palmero I Ortiz-Rodriguez A Ghorbanpoor S Kucharski LC. Dehydroepiandrosterone protects male and female hippocampal neurons and neuroblastoma cells from glucose deprivation. Brain Res. (2016) 1644:176–82. 10.1016/j.brainres.2016.05.014 27174000

[111] Mesiano S Jaffe RB. Developmental and functional biology of the primate fetal adrenal cortex. Endocr Rev. (1997) 18, 378–403. 10.1210/edrv.18.3.0304 9183569

[112] Bernstein RM Sterner KN Wildman DE. Adrenal androgen production in catarrhine primates and the evolution of adrenarche. Am J Phys Anthropol. (2012) 147:389–400. 10.1002/ajpa.22001 22271526 PMC4469270

[113] Nguyen AD Mapes SM Corbin CJ Conley AJ. Morphological adrenarche in rhesus macaques: development of the zona reticularis is concurrent with fetal zone regression in the early neonatal period. J Endocrinol. (2008) 199:367–78. 10.1677/JOE-08-0337 18787057

[114] Conley AJ Plant TM Abbott DH Moeller BC Stanley SD. Adrenal androgen concentrations increase during infancy in male rhesus macaques (Macaca mulatta). Am J Physiol Endocrinol Metab. (2011) 301:E1229–35. 10.1152/ajpendo.00200.2011 21900126 PMC3274962

[115] Muehlenbein MP Campbell BC Richards RJ Svec F Phillippi-Falkenstein KM Murchison MA. Dehydroepiandrosterone-sulfate as a biomarker of senescence in male non-human primates. Exp Gerontol. (2003) 38:1077–85. 10.1016/j.exger.2003.07.001 14580861

[116] Krøll J. (2015). Dehydroepiandrosterone, molecular chaperones and the epigenetics of primate longevity. Rejuvenation Res. (2015) 18:341–6. 10.1089/rej.2014.1641 25706901

[117] Pattison JC Saltzman W Abbott DH Hogan BK Nguyen AD Husen B Gender and gonadal status differences in zona reticularis expression in marmoset monkey adrenals: cytochrome b5 localization with respect to cytochrome P450 17,20-lyase activity. Mol Cell Endocrinol. (2007) 265–6:93–101. 10.1016/j.mce.2006.12.023 PMC1839875 17222503

[118] Pattison JC Abbott DH Saltzman W Conley AJ Bird IM. Plasticity of the zona reticularis in the adult marmoset adrenal cortex: voyages of discovery in the new world. J Endocrinol. (2009) 203:313–26. 10.1677/JOE-08-0554 19474057

[119] Kushnir MM Blamires T Rockwood AL Roberts WL Yue B Erdogan E. Liquid chromatography-tandem mass spectrometry assay for androstenedione, dehydroepiandrosterone, and testosterone with pediatric and adult reference intervals. Clin Chem. (2010) 56:1138–47. 10.1373/clinchem.2010.143222 20489135

[120] Remer T Boye KR Hartmann MF Wudy SA. Urinary markers of adrenarche: reference values in healthy subjects, aged 3-18 years. J Clin Endocrinol Metab. (2005) 90:2015–21. 10.1210/jc.2004-1571 15671100

[121] Langergraber KE Prüfer K Rowney C Boesch C Crockford C Fawcett K. Generation times in wild chimpanzees and gorillas suggest earlier divergence times in great ape and human evolution. Proc Natl Acad Sci USA. (2012) 109:15716–21. 10.1073/pnas.1211740109 22891323 PMC3465451

[122] Copeland KC Eichberg JW Parker CR Jr Bartke A. Puberty in the chimpanzee: somatomedin-C and its relationship to somatic growth and steroid hormone concentrations. J Clin Endocrinol Metabol. (1985) 60:1154–60. 10.1210/jcem-60-6-1154 3158669

[123] Wudy SA Hartmann MF Remer T. Sexual dimorphism in cortisol secretion starts after age 10 in healthy children: urinary cortisol metabolite excretion rates during growth. Am J Physiol Endocrinol Metab. (2007) 293:E970–6. 10.1152/ajpendo.00495.2006 17638704

[124] Houghton LC Cooper GD Booth M Chowdhury OA Troisi R Ziegler RG. Childhood environment influences adrenarcheal timing among first-generation Bangladeshi migrant girls to the UK. PLoS ONE. (2014) 9:e109200. 10.1371/journal.pone.0109200 25309977 PMC4195659

[125] Utriainen P Laakso S Liimatta J Jääskeläinen J Voutilainen R. Premature adrenarche–a common condition with variable presentation. Hormone Res Paediatrics. (2015) 83:221–31. 10.1159/000369458 25676474

[126] Henrich J Heine SJ Norenzaya A. The weirdest people in the world. Behav Brain Sci. (2010) 33:61–83. 10.1017/S0140525X0999152X 20550733

[127] Shi L Wudy SA Buyken AE Hartmann MF Remer T. Body fat and animal protein intakes are associated with adrenal androgen secretion in children. Am J Clin Nutr. (2009) 90:1321–8. 10.3945/ajcn.2009.27964 19793857

[128] Mavoungou D Gass R Emane MN Cooper RW Roth-Meyer C. Plasma dehydroepiandrosterone, its sulfate, testosterone and FSH during puberty of African children in Gabon. J Steroid Biochem. (1986) 24:645–51. 10.1016/0022-4731(86)90132-9 2939299

[129] Tung YC Lee JS Tsai WY Hsiao PH. Physiological changes of adrenal androgens in childhood. J Formosa Med Assoc. (2004) 103:921–4. 15624041

[130] Campbell BC Mbivzo MT. Testosterone, reproductive maturation and somatic growth among Zimbabwe Boys. Ann Hum Biol. (2006) 33:17–25. 10.1080/03014460500424068 16500808

[131] Walker R Gurven M Hill K Migliano A Chagnon N de Souza R. Growth rates and life histories in twenty-two small-scale societies. Am J Hum Biol. (2006) 18:295–311. 10.1002/ajhb.20510 16634027

[132] Crittenden AN Schnorr SL. Current views on hunter-gatherer nutrition and the evolution of the human diet. Am J Phys Anthropol. (2017) 162(Suppl. 63):84–109. 10.1002/ajpa.23148 28105723

[133] Martin M Blackwell A Gurven M Kaplan H Make new friends and keep the old? parasite coinfection and comorbidity in homo sapiens In: Brinkworth JF Pechenkina K editors. Primates, Pathogens, and Evolution. New York, NY: Springer (2013). p. 363–87. 10.1007/978-1-4614-7181-3_12

[134] Parker CR Jr Grizzle WE Blevins JK Hawkes K. Development of adrenal cortical zonation and expression of key elements of adrenal androgen production in the chimpanzee (*Pan troglodytes*) from birth to adulthood. Mol Cell Endocrinol. (2014) 387:35–43. 10.1016/j.mce.2014.02.010 24576611 PMC4016767

[135] Smith TM Machanda Z Bernard AB Donovan RM Papakyrikos AM Muller MN. First molar eruption, weaning, and life history in living wild chimpanzees. Proc Natl Acad Sci USA. (2013) 110:2787–91. 10.1073/pnas.1218746110 23359695 PMC3581971

[136] Machanda Z Brazeau NF Bernard AB Donovan RM Papakyrikos AM Wrangham R. Dental eruption in East African wild chimpanzees. J Hum Evol. (2015) 82:137–44. 10.1016/j.jhevol.2015.02.010 25796539

[137] Smith H Life history and the evolution of human maturation. Evol Anthropol. (1992) 1:134–42. 10.1002/evan.1360010406

[138] Bauernfeind AL Barks SK Duka T Grossman LI Hof PR Sherwood CC. Aerobic glycolysis in the primate brain: reconsidering the implications for growth and maintenance. Brain Struct Funct. (2014) 219:1149–67. 10.1007/s00429-013-0662-z 24185460

[139] Bogin B Bragg J Kuzawa C Childhood, biocultural reproduction and human lifetime reproductive effort. In: Meheen CL Crittenden A editors. Childhood: Origins, Evolution and Implications. Albuquerque: University New Mexico/SAR Press (2016). p. 45–72.

[140] Kramer KL. The evolution of human parental care and recruitment of juvenile help. Trends Ecol Evol. (2011) 26:533–40. 10.1016/j.tree.2011.06.002 21784548

[141] Kramer KL Otárola-Castillo E. When mothers need others: the impact of hominin life history evolution on cooperative breeding. J. Hum. Evol. (2015) 84:16–24. 10.1016/j.jhevol.2015.01.009 25843884

[142] Thompson JL Nelson AJ Childhood and patterns of growth in the genus homo. In: Meehan CL Crittenden A editors. Childhood: Origins, Evolution and Implications. Santa Fe: University of New Mexico Press/School of American Research (2016). p. 75–102.

[143] Meheen CL Crittenden A editors. Childhood: Origins, Evolution and Implications. Albuquerque, NM: University of New Mexico/SAR Press (2016).

[144] Weisner TS Gallimore K My brother's keeper: child and sibling caretaking. Curr Anthropol. (1977) 18:169–90. 10.1086/201883

[145] Xiao Y Geng F Riggins T Chen G Redcay E. Neural correlates of developing theory of mind competence in early childhood. Neuroimage. (2019) 184:707–16. 10.1016/j.neuroimage.2018.09.079 30273714 PMC6230512

[146] Call J Tomasello M. Does the chimpanzee have a theory of mind? 30 years later. Trends Cogn Sci. (2008) 12:187–192. 10.1016/j.tics.2008.02.010 18424224

[147] Gweon H Dodell-Feder D Bedny M Saxe R. Theory of mind performance in children correlates with functional specialization of a brain region for thinking about thoughts. Child Dev. (2012) 83:1853–68. 10.1111/j.1467-8624.2012.01829.x 22849953

[148] Saxe R. Uniquely human social cognition. Curr Opin Neurobiol. (2006) 16:235–9. 10.1016/j.conb.2006.03.001 16546372

[149] Miller KK Al-Rayyan N Ivanova MM Mattingly KA Ripp SL Klinge CM. DHEA metabolites activate estrogen receptors alpha and beta. Steroids. (2013) 78:15–25. 10.1016/j.steroids.2012.10.002 23123738 PMC3529809

[150] Barendse MEA Simmons JG Byrne ML Seal ML Patton G Mundy L. Brain structural connectivity during adrenarche: associations between hormone levels and white matter microstructure. Psychoneuroendocrinology. (2018) 88:70–7. 10.1016/j.psyneuen.2017.11.009 29175736

[151] Friedrich N Völzke H Rosskopf D Steveling A Krebs A Nauck M. Reference ranges for serum dehydroepiandrosterone sulfate and testosterone in adult men. J Androl. (2008) 29:610–7. 10.2164/jandrol.108.005561 18599883

[152] Sulcová J Hill M Hampl R Stárka L. Age and sex related differences in serum levels of unconjugated dehydroepiandrosterone and its sulphate in normal subjects. Endocrinology. (1997) 154:57–62. 10.1677/joe.0.1540057 9246938

[153] Gogtay N Giedd JN Lusk L Hayashi KM Greenstein D Vaituzis AC. Dynamic mapping of human cortical development during childhood through early adulthood. Proc Natl Acad Sci USA. (2004) 101:8174–9. 10.1073/pnas.0402680101 15148381 PMC419576

[154] Shaw P Kabani NJ Lerch JP Eckstrand K Lenroot R Gogtay N. Neurodevelopmental trajectories of the human cerebral cortex. J Neurosci. (2008) 28:3586–94. 10.1523/JNEUROSCI.5309-07.2008 18385317 PMC6671079

[155] Westlye LT Walhovd KB Dale AM Bjørnerud A Due-Tønnessen P Engvig A. Differentiating maturational and aging-related changes of the cerebral cortex by use of thickness and signal intensity. Neuroimage. (2010) 52:172–185. 10.1016/j.neuroimage.2010.03.056 20347997

[156] Hockberg Z Konner M Emerging adulthood: a pre-adult life-history stage. Front Endocrinol. (2020) 10:918 10.3389/fendo.2019.00918 PMC6970937 31993019

[157] Miller DJ Duka T Stimpson CD Schapiro SJ Baze WB McArthur MJ. Prolonged myelination in human neocortical evolution. Proc Natl Acad Sci USA. (2012) 109:16480–5. 10.1073/pnas.1117943109 23012402 PMC3478650

[158] Hoekzema E Barba-Müller E Pozzobon C Picado M Lucco F García-García D. Pregnancy leads to long-lasting changes in human brain structure. Nat Neurosci. (2017) 20:287–96. 10.1038/nn.4458 27991897

[159] Hewlett BS Lamb ME editors. Hunter-Gatherer Childhoods: Evolutionary, Developmental and Cultural Perspectives. New Brunswick, NJ: Taylor and Francis (2006).

[160] Froehle AW Wells GK Pollom TR Mabulla AZP Lew-Levy S Crittenden AN. Physical activity and time budgets of Hadza forager children: Implications for self-provisioning and the ontogeny of the sexual division of labor. Am J Hum Biol. (2019) 31:e23209. 10.1002/ajhb.23209 30576026 PMC6342658

[161] Liebenberg L The Art of Tracking: The Original Science. Capetown: David Phillips (2012).

[162] Gutiérrez-Castellanos N Martínez-Marcos A Martínez-García F Lanuza E. Chemosensory function of the amygdala. VitamHorm. (2010) 83:165–96. 10.1016/S0083-6729(10)83007-9 20831946

[163] Patin A Pause BM. Human amygdala activations during nasal chemoreception. Neuropsychologia. (2015) 78:171–94. 10.1016/j.neuropsychologia.2015.10.009 26459095

[164] Joannes-Boyau R Justin W Adams JW Austin C Arora M Moffat I. Elemental signatures of *Australopithecus* africanus teeth reveal seasonal dietary stress. Nature. (2019) 572:112–5. 10.1038/s41586-019-1370-5 31308534 PMC7359858

[165] Spoor F Gunz P Neubauer S Stelzer S Scott N Kwekason A. Reconstructed homo habilis type OH 7 suggests deep-rooted species diversity in early Homo. Nature. (2015) 519:83–6. 10.1038/nature14224 25739632

[166] Plummer T Flaked stones and old bones: biological and cultural evolution at the dawn of technology. Am J Phys Anthropol. (2004) 125(Suppl. 39):118–64. 10.1002/ajpa.20157 15605391

[167] Ungar PS Grine FE Teaford MF El Zaatari S. Dental microwear and diets of African early Homo. J Hum Evol. (2006) 50:78–95. 10.1016/j.jhevol.2005.08.007 16226788

[168] Carmody RN Wrangham RW. The energetic significance of cooking. J Hum Evol. (2009) 57:379–91. 10.1016/j.jhevol.2009.02.011 19732938

[169] Roebroeks W Villa P. On the earliest evidence for habitual use of fire in Europe. Proc Natl Acad Sci USA. (2011) 108:5209–14. 10.1073/pnas.1018116108 21402905 PMC3069174

[170] Shimelmitz R Kuhn SL Jelinek AJ Ronen A Clark AE Weinstein-Evron M. 'Fire at will': the emergence of habitual fire use 350,000 years ago. J Hum Evol. (2014) 77:196–203. 10.1016/j.jhevol.2014.07.005 25439628

[171] Cornélio AM de Bittencourt-Navarrete RE de Bittencourt-Brum R Queiroz CM Costa MR. Human brain expansion during evolution is Independent of fire control and cooking. Front Neurosci. (2016) 10:167. 10.3389/fnins.2016.00167 27199631 PMC4842772

[172] Cunnane SC Crawford MA. Energetic and nutritional constraints on infant brain development: implications for brain expansion during human evolution. J Hum Evol. (2014) 77:88–98. 10.1016/j.jhevol.2014.05.001 24928072

[173] Thompson JC Carvalho S Curtis W Marean CW Alemseged Z Origins of the human predatory pattern: the transition to large-animal exploitation by early hominins. Curr Anthropol. (2019) 60:1–23. 10.1086/701477

[174] Pereira PM Vicente AF. Meat nutritional composition and nutritive role in the human diet. Meat Sci. 93:586–92. 10.1016/j.meatsci.2012.09.018 23273468

[175] Ellison PT. Endocrinology, energetics and human life history: a synthetic model. Horm Behav. (2017) 91:97–106. 10.1016/j.yhbeh.2016.09.006 27650355

[176] Wilson EO Consilience: The Unity of Knowledge. New York, NY: Knopf (1998).

[177] de Fockert JW Caparos S Linnell KJ Davidoff J. Reduced distractibility in a remote culture. PLoS ONE. (2011) 6:e26337. 10.1371/journal.pone.0026337 22046275 PMC3198475

[178] Lancy DF Ethnographic perspectives on culture acquisition. In: Meehan CL Crittenden A editors. Childhood: Origins, Evolution and Implications. University of New Mexico/SAR Press (2016). p. 173–95.

[179] Sanou AS Diallo AH Holding P Nankabirwa V Engebretsen IMS Ndeezi G. Effects of schooling on aspects of attention in rural Burkina Faso, West Africa. PLoS ONE. (2018) 13:e0203436. 10.1371/journal.pone.0203436 30183764 PMC6124811

[180] Linnell KJ Caparos S de Fockert JW Davidoff J. Urbanization decreases attentional engagement. J Exp Psychol Hum Percept Performance. (2013) 39:1232–47. 10.1037/a0031139 23339348

[181] Bird-David N “Animism” revisited: personhood, environment and relational epistemology w/ comments. Curr Anthropol. (1999) 40: S67–91. 10.1086/200061

[182] Guenther M Tricksters & Trancers: Bushman Religion and Society. Bloominton: Indiana University Press (1999).

[183] Keeney B Keeney H Reentry into first creation: a contextual frame for the Ju/'hoan bushman performance of puberty rites, storytelling, and healing dance. J Anthropol Res. (2013) 69:65–85. 10.3998/jar.0521004.0069.104

[184] Hallowell I Ojibwa ontology, behavior and world view. In: Diamond S editor. Culture in History: Essays in Honor of Paul Radin. New York, NY: Columbia University Press (1960). p. 141–79.

